# Optimizing Allocation to Telehealth and In-Person Prolonged Exposure for Women Veterans with Military Sexual Trauma: A Precision Medicine Approach

**DOI:** 10.3390/bs14110993

**Published:** 2024-10-24

**Authors:** Evangelia Argyriou, Daniel F. Gros, Melba A. Hernandez Tejada, Wendy A. Muzzy, Ron Acierno

**Affiliations:** 1 Department of Psychiatry, Indiana University School of Medicine, Indianapolis, IN 46202, USA; evargyri@iu.edu; 2 Mental Health Service, Ralph H. Johnson VA Healthcare System, Charleston, SC 29401, USA; muzzy@musc.edu (W.A.M.); ronald.acierno@uth.tmc.edu (R.A.); 3 Department of Psychiatry & Behavioral Sciences, Medical University of South Carolina, Charleston, SC 29425, USA; 4 Faillace Department of Psychiatry, University of Texas Health Science Center at Houston, Houston, TX 77030, USA; melba.a.hernandeztejada@uth.tmc.edu

**Keywords:** precision medicine, individualized treatment rule, PTSD, military sexual trauma, prolonged exposure, telehealth

## Abstract

Military sexual trauma-related post-traumatic stress disorder (PTSD) is highly prevalent and costly among women veterans, making the need for effective and accessible treatment of critical importance. Access to care is a key mechanism of mental health disparities and might affect differential response to treatment. The goal of this study was to estimate an individualized treatment rule based on readily available individual characteristics related to access to care to optimize allocation to in-person vs. telehealth delivery of prolonged exposure for PTSD in military sexual trauma survivors. The following variables were used as prescriptive factors: age, race, disability status, socioeconomic status, rural vs. urban status, and baseline PTSD level. The rule was estimated using a machine-learning approach, Outcome Weighted Learning. The estimated optimal rule outperformed a one-size-fits-all rule where everyone is universally assigned to telehealth; it led to markedly lower mean PTSD levels following 6 months from treatment (Vd_opt_ − V_Telehealth_ = −14.55, 95% CI: −27.24, −1.86). However, the rule did not significantly discriminate for in-person therapy (Vd_opt_ − V_In-person_ = −11.86, 95% CI: −25.83, 2.12). Upon further validation with larger and more diverse samples, such a rule may be applied in practice settings to aid clinical decision-making and personalization of treatment assignment.

## 1. Introduction

### 1.1. Military Sexual Trauma and Post-Traumatic Stress Disorder

Military sexual trauma (MST) involves experiencing sexual assault or threatening sexual harassment during military service [1]. This includes any sexual activity against a person’s will or when they are unable to say no during military service. MST is highly prevalent among veterans, especially women, with 38.4% having experienced harassment and/or assault, according to a meta-analysis [2]. MST has been linked to a range of psychological, physical, social, and occupational difficulties, including post-traumatic stress disorder (PTSD; a psychiatric disorder that may occur in response to experiencing or witnessing a traumatic event and involves symptoms that fall into four classes: intrusion, avoidance, alterations in cognition and mood, and alterations in arousal and reactivity) [3,4]. A history of MST is also associated with higher severity of clinical presentation, including increased comorbid mental health conditions [5,6,7,8,9,10]. Other costs associated with MST and MST-related PTSD include physical problems, such as chronic pain, menstrual problems, and chronic fatigue, as well as other effects on functioning such as difficulty maintaining employment, reduced quality of life, income disparities, and family and relationship difficulties [11,12,13,14].

Importantly, women veterans who experienced MST are up to nine times more likely to develop PTSD compared to those without a history of MST [4,15]. Moreover, while male veterans generally have shown a higher prevalence of PTSD compared to female veterans, the link between MST and PTSD diagnoses seems more pronounced in women [16]. In addition to high rates and prevalence of MST-related PTSD among women, women with MST-related PTSD [17,18] have higher dropout rates than those with combat-related PTSD [19], which shows that even when they receive treatment, it is less successful. Therefore, addressing MST-related PTSD effectively is crucial for active-duty and veteran women.

### 1.2. Psychotherapy for MST-Related PTSD

Given the significant costs of MST-related PTSD, providing effective and accessible treatment is of critical importance [16]. Several evidence-based psychotherapies exist with validation in active-duty military or veteran populations and are recommended as first-line treatments for PTSD [19]. One of these is prolonged exposure (PE). PE is an intensive, manualized psychotherapy typically offered in specialty mental health settings and has strong evidence of effectiveness in reducing PTSD as well as depressive symptoms in civilian and veteran populations [20,21,22,23,24,25]. PE involves imaginal exposure to the traumatic event with the goal of extinguishing fear responses associated with conditioned traumatic memory cues, and in vivo exposure to triggers and reminders of the event.

Despite the evidence of support and the wide implementation of evidence-based psychotherapies for PTSD, MST-related service utilization remains low, with about one in two among those reporting MST being engaged in MST-related care [26]. Even among those who seek treatment, treatment dropout is considerably high and even higher than among PTSD treatment seekers in general, with dropout rates ranging between 40 and 60% [17,18]. A wide range of barriers to care has been identified that might lead to low treatment utilization. Among women veterans with MST-related PTSD, systemic barriers to care might be particularly relevant to this low treatment utilization and treatment adherence. The Veterans Affairs Medical Centers are primarily male-dominated context, which might deter women veterans with MST-related PTSD from engaging in treatment [16]. In addition, basic logistical barriers include difficulty attending consecutive multiple weekly appointments, parking issues, travel time, and lost work time, particularly for those commuting from rural areas or for primary caregivers of young children [27,28,29].

### 1.3. Telehealth as an Alternative to In-Person Treatment

Such barriers can be addressed by delivering psychotherapy via home telehealth to bolster access to care. Accumulating research supports the efficacy of telehealth-based psychotherapy for various evidence-based treatments [30,31,32] and PE specifically [16,33]. Especially for the population of women veterans with MST-related PTSD, providing telehealth-based PE, in theory, could address the issue of accessibility and lead to improved outcomes compared to in-person PE. However, previous research has not provided evidence of the superiority of this modality for this or other populations [30,33,34]. The lack of difference, on average, between the two treatment modalities could indicate their equivalence for individuals. However, this overall equivalence may mask heterogeneity in treatment response between the two modalities across different subgroups. In other words, treatment accessibility and, by connection, treatment completion may look very different among population subgroups who face different systemic barriers to care.

### 1.4. Personalized Assignment to In-Person and Telehealth Modalities

Addressing heterogeneity in response to in-person versus telehealth can optimize allocation to the two modalities and maximize clinical outcomes overall. Heterogeneity in response to treatment is well-documented in the literature [35,36,37,38] and illustrates a need for personalized, data-driven approaches to clinical decision-making. Precision medicine approaches for personalized treatment selection can address this heterogeneity and leverage individual characteristics to optimize treatment allocation based on individual needs [39,40]. One means to accomplish this is by estimating individualized treatment rules. Individualized treatment rules are data-driven decision rules that take available individual characteristics (i.e., prescriptive factors) as input and yield a recommendation of the optimal treatment choice among available options based on which of the options is expected to benefit a given individual the most [41,42,43]. Individualized treatment rules can then be implemented in clinical settings to assist clinical decision-making.

Previous research has developed and tested such rules with promising results. Many of these studies have focused on optimal medication allocation. For example, Kessler et al. (2022) [44] developed an individualized treatment rule to optimize second-line antidepression medication selection to maximize the probability of remission after failing a first-line antidepressant in a sample of previously untreated patients with major depressive disorder. Their findings showed that assignment according to the rule was associated with increased remission rates compared to randomization (i.e., not using the rule). Other studies applied this approach to optimize allocation among psychotherapies. Argyriou et al. (2024) [45] estimated an individualized treatment rule using a machine-learning approach to optimize assignment to two evidence-based interventions for grief: behavioral activation and therapeutic exposure, and cognitive therapy for grief. They found that assigning participants according to this rule led to ~18–20-point lower mean grief levels at six months post-treatment compared to assigning everyone universally to either psychotherapy regardless of their characteristics (i.e., not using the rule). Estimating an individualized treatment rule for optimal allocation among telehealth and in-person PE modalities might improve outcomes compared to universally recommending one over the other or completely relying on client preferences.

### 1.5. Potential Contributors to Differential Response to In-Person Versus Telehealth PE

Access to care is an important mechanism of mental health disparities and might affect differential response to treatment [46]. Addressing mechanisms of disparities, such as differential access to care, can advance mental health equity and thus improve mental health outcomes. Optimizing allocation to in-person versus telehealth modalities based on individual characteristics that predict differential access to care might be a first step toward achieving this goal. A number of variables may contribute to increased telehealth benefit compared to in-person PE. Geographic location may be one of these. Underserved rural populations typically have shortages of mental health services in their communities [47,48]. Research has shown that telehealth services reduce gaps in the overall rural–urban difference in specialty care use, although this gap has not been eliminated [49]. People with disabilities may also experience similar barriers to reach in-person services and benefit from high-quality care [50,51]. Telehealth may eliminate well-documented transportation barriers for individuals with mobility limitations [52].

On the other hand, other characteristics may relate to higher benefits from in-person therapy as opposed to telehealth. Using telehealth requires digital literacy and the ability to adapt to changing technology. Therefore, mental health services through videoconferencing might be less beneficial for older adults with lower digital literacy [53,54]. Relatedly, previous research demonstrated that older adults were less likely to express willingness to use telehealth as a treatment modality [55]. Despite this, some recent evidence shows that a growing number of older adults are accepting telehealth services [56]. For instance, a study involving 618,000 older adults found that older adults were generally open to adopting telehealth, indicating a positive trend toward acceptance and adaptation [57]. Among positive points: easier access to care, particularly for those with mobility issues or living in remote areas. Reduced preference for telehealth was also observed among Black individuals compared to other racial/ethnic groups and among those with disadvantaged socioeconomic backgrounds [55]. In a national sample of 14,305,819 US Medicare enrollees, taking into account geographic location, non-white racial groups received fewer telehealth visits than white individuals [58] (although other studies have found no differences in telehealth visit attendance across race [59]). Access to or good quality broadband internet might be limited in populations with financial constraints, which makes telehealth options impossible or less effective [54,60]. Therefore, for these subgroups, in-person treatment might involve fewer barriers compared to telehealth. Fewer barriers to treatment are expected to lead to a better response to treatment overall. Taking into account individual characteristics potentially associated with such barriers when making decisions about recommending telehealth versus in-person services may maximize benefit from PE overall.

### 1.6. Current Study

In the present study, we conducted a secondary analysis of a randomized clinical trial comparing in-person vs. home-based telehealth delivery of PE for PTSD in women military sexual trauma survivors [16] to estimate an individualized treatment rule for optimal allocation among the two modalities. In this study, women veterans with MST-related PTSD were randomized to identical PE treatment delivered either via telehealth or in-person. Treatment occurred weekly over 12–15 weeks in 90-min sessions. Initial hypotheses of the original study predicted that women in the telehealth PE group would complete more sessions and show greater reductions in PTSD and depression symptoms compared to in-person PE. However, no differences were found between conditions. The lack of difference, on average, between the two treatment modalities could indicate their equivalence. On the other hand, this overall equivalence may mask heterogeneity in treatment response among the two modalities across different subgroups.

To address this possibility, we used a machine-learning-based approach to estimate an individualized treatment rule to optimize allocation to the two PE modalities based on a set of readily available baseline characteristics that might differentially relate to treatment response as suggested by the literature [47,48,49,50,51,52,53,54,55,60]. Specifically, we used the following variables as potential prescriptive factors: age, race, disability status, socioeconomic status, rural vs. urban status, and baseline PTSD level. We hypothesized that the lack of difference between the two treatment modalities is a result of the heterogeneity in treatment response among different population subgroups. We expected that taking into account these individual characteristics in treatment assignment would improve PTSD outcomes six months after treatment overall. Thus, the optimal individualized treatment rule would perform better compared to one-size-fits-all rules where everyone is universally assigned to a given modality.

## 2. Materials and Methods

### 2.1. Participants

Participants were 122 women veterans, out of 172 initially screened for eligibility, with MST-related PTSD who were recruited from a VAMC in the Southeastern United States and randomly assigned to either a telehealth PE condition (N = 62) or an in-person PE (N = 60) condition. Eligibility criteria were as follows: (1) an MST-related index event as identified on the Stressful Events for Veterans Questionnaire, which was adapted for veterans based on the National Stressful Events Survey and MST screener questions [16,61,62]; and (2) meeting criteria for PTSD related to MST based on the Clinician Administered PTSD Scale (CAPS-5; Weathers et al., 2018 [63]). Participants were excluded if they screened positive for an active psychotic episode, major neurocognitive disorder, severe substance use disorder, or endorsed active suicidal ideation with clear intent. Other exclusion criteria were concurrent enrollment in a clinical trial for PTSD or depression and participation of another household member in our study. Participants were also excluded if they had not completed the baseline assessment. If participants had a recent change to their psychotropic medication, a period of four weeks for medication stabilization was required before enrolling in this study.

### 2.2. Treatment Conditions

The two PE conditions were identical with the exception of the modality used (i.e., in-person vs. telehealth). PE involves 12–15 90-min sessions administered weekly [64]. The main components of this treatment are psychoeducation about common reactions to trauma, symptom maintenance and the function of PE, in vivo exposure to avoided conditioned traumatic stimuli, and imaginal exposure to the traumatic memory. PE has strong evidence of support for its effectiveness and is one of the first-line recommended treatments for veterans with PTSD [21,64,65,66,67].

### 2.3. Measures

Demographic characteristics. Participants were asked to report their gender (the final sample included only women), age, and race (White, Black, Native American or Alaskan Native, Asian, Pacific Islander or Native Hawaiian, more than one race, or other), total annual combined income, and disability status. Rural status was determined using the participants’ zip codes. The geographic location was classified as rural for locations with fewer than 500 persons per square mile, as defined by the USDA. Annual household income was used as a proxy for SES. Participants were asked to select the category to which their annual household income belonged (USD 0–<USD 10,000, USD 10,000–<USD 15,000, USD 15,000–<USD 20,000, USD 20,000–<USD 25,000, USD 25,000–<USD 35,000, USD 35,000–<USD 50,000, USD 50,000–<USD 75,000, USD 75,000 or more).

PTSD Checklist for Diagnostic and Statistical Manual of Mental Disorders-fifth edition (PCL-5). The PCL-5 is a self-report measure of PTSD symptoms based on the DSM-5 criteria for PTSD. Participants are asked to rate how much they were bothered by a list of problems in the past month based on a 5-point scale (from 0 = Not at all to 4 = Extremely). The total score ranges from 0 to 80. This measure has been validated in veteran samples and has shown good psychometric properties [68]. Cronbach’s alpha in our sample was 0.89. The PCL-5 was measured at baseline which was included as a prescriptive factor in the main analysis, post-treatment, and six months following treatment completion. The 6-month PCL-5 score was used as the main outcome of interest. We focused on this time point because our primary goal was to maximize long-term benefits from treatment instead of immediate effects that could be transient.

Clinician Administered PTSD Scale (CAPS-5). The CAPS-5 was used to determine whether participants met criteria for PTSD related to MST. The CAPS-5 is a structured diagnostic interview for PTSD [63]. The CAPS-5 assesses PTSD symptom criteria and related features such as dissociation. The CAPS-5 and the previous version of the CAPS have been extensively validated in different samples, including veterans [63,69]. The CAPS-5 has shown strong psychometric properties, including interrater reliability, test-retest reliability, and convergent and discriminant validity [63].

### 2.4. Procedures

A detailed description of the recruitment procedures can be found in Acierno et al. (2021) [16]. All procedures were approved by the affiliate University Internal Review Board and the partnering VAHCS. Participants were informed about the study through the local MST Care Coordination Team, PTSD Clinical Team, VA primary care, and other local VA clinics. All referrals completed consent documentation during the intake. The project coordinator randomized qualifying participants to telehealth or in-person PE using REDCap. Generated assignments were reviewed by the statistician, and thus no changes to the assignments could be made. Assessments were conducted in-person (for baseline and post-treatment) and by telephone (3- and 6-month follow-ups). Assessors were blinded to the treatment condition to which participants were assigned. Participants were compensated up to USD 180.00 for their participation in the study.

### 2.5. Statistical Analysis

Data management and statistical analyses were conducted using the R statistical software v. 4.0.3 (R Project for Statistical Computing, Vienna, Austria). Data were first inspected for entry errors and outliers for quality assurance using summary statistics and visualizations. Variables were standardized before the main analysis (i.e., rescaled to have a mean of zero and a standard deviation [SD] of one). Due to the low number of participants identifying with a race other than Black or White (N = 9), race was converted into the binary variable white (0 = no; 1 = yes). For annual household income, a binary variable for lower income (0 = no, 1 = yes) was created. The lower-income category included incomes in the lowest quintile (20th percentile) based on data from the U.S. Census. The threshold was <USD 35,000, which includes incomes estimated to be in the lowest percentile for all years that the data collection was active (2016–2019) [70,71,72,73,74]. Descriptive statistics for study variables were examined. Means and SD were examined for continuous variables, and frequencies and percentages for categorical variables.

#### Estimation and Performance of the Optimal Individualized Treatment Rule

To optimize assignment between in-person and telehealth PE, we estimated an optimal individualized treatment rule using Outcome Weighted Learning [75]. This method is based on machine-learning and causal inference frameworks and uses a weighted version of support vector machines to estimate an optimal decision function for differential treatment assignment [45]. The decision rule is estimated directly (as opposed to other two-step approaches) using a weighted classification framework that maximizes response to treatment. This is a flexible, nonparametric approach with minimal assumptions compared to parametric linear regression-based methods.

The performance of the optimal individualized rule was assessed by estimating the value of the outcome (6-month PCL-5 score) under the rule (Vd_opt_) and comparing it to one-size-fits-all rules that universally assign everyone to in-person PE (Vd_opt_ − V_in-person_) or telehealth PE (Vd_opt_ − V_telehealth_). To address the possibility of overfitting (i.e., the optimal rule not being generalizable outside of the current sample), we employed the following procedures (for more details, see [45]). We used L_2_ (ridge) penalization to penalize the complexity of the decision function (i.e., shrinking coefficient values toward zero). The Jackknife approach (leave-one-out cross-validation) was used to select the optimal penalty parameter and evaluate the out-of-sample performance of the estimated optimal rule [76]. In this approach, a single individual serves as a fold, and *n* − 1 folds (individuals) are utilized for training the algorithm, while the excluded fold is used for testing the algorithm. This process is repeated until each fold has been utilized for testing.

An intention-to-treat analysis was used with all participants included regardless of treatment completion status. This way the estimated rule would not be biased toward treatment completers. Inverse probability weighting was used to account for potential selection bias from missing PCL-5 outcome values [77]. The covariates used to predict the probability of missingness in this approach included all the prescriptive variables, in addition to the treatment condition. Standard error estimation was based on the bootstrap with 1000 replications.

## 3. Results

### 3.1. Descriptive Statistics

Means, SD, and frequencies of study characteristics, overall and by treatment condition, as well as the percentage of missingness for each variable, are presented in Table 1. None of the variables differed as a function of the treatment condition to which participants were randomized, except for age. Those assigned to in-person PE were older by about five years on average (*p* = 0.037). There was no overall significant difference in PCL-5 score following 6 months from treatment between telehealth and in-person PE (*t* = −0.65, *p* = 0.519).

### 3.2. The Estimated Individualized Treatment Rule and Impact on Overall PTSD Symptom Level

The estimated optimal individualized treatment rule assigned participants to two subgroups based on the estimated benefit or harm from each treatment modality. Fifty-four participants were assigned to telehealth PE, and 59 were assigned to in-person PE. The estimated individualized treatment rule was:

d_opt_(*X*) = Ι(−0.23 − 0.84 × ***Age***
*−* 0.96 × ***White*** + 0.02 × ***Disability***

+ 0.08 × ***Rural*** + 0.63 × ***SES***
*−* 0.02 × ***Baseline PCL-5*** ≥ 0)

where

d_opt_(*X*) = the estimated optimal assignment rule to be used on a future individual, member of the target population

*I*(score ≥ 0) = indicator function which is equal to 1 (i.e., telehealth PE) if score ≥ 0 and 0 (i.e., in-person PE) if score < 0

Values = the estimated decision coefficients

Variables in bold *=* prescriptive variables. These will be replaced with measured values of a future individual, member of the target population.

The mean PCL-5 score under the estimated individualized treatment rule (i.e., by assigning participants to the treatment expected to benefit them the most based on their baseline characteristics) was 17.83 (95% CI: 3.90, 31.76; see Table 2). The optimal rule performed significantly better than assigning everyone to telehealth since assigning participants to the optimal treatment for them based on their characteristics led to markedly lower mean PCL-5 scores following 6 months from treatment compared to assigning everyone universally to telehealth PE (Vd_opt_ − V_Telehealth_ = −14.55, 95% CI: −27.24, −1.86). The optimal rule led to a lower mean PCL-5 score compared to the one-size-fits-all rule assigning everyone to in-person PE, although this difference was not statistically significant (Vd_opt_ − V_In-person_ = −11.86, 95% CI: −25.83, 2.12).

## 4. Discussion

The goal of this study was to estimate an optimal individualized treatment rule using a machine-learning method to optimize assignment between in-person and telehealth PE among women veterans with MST-related PTSD. Our results showed that an individualized rule leveraging simple and easily assessed individual characteristics in clinical practice outperforms a one-size-fits-all rule where everyone is universally assigned to telehealth. No significant difference was found between assignment by the rule and the one-size-fits-all rule where everyone is universally assigned to in-person PE.

The finding that the estimated individualized treatment rule led to significantly lower PTSD symptom levels compared to assigning everyone to telehealth provides preliminary evidence against the equivalence of the two treatment modalities of PE. Previous research examining average treatment effects has shown no evidence of superiority of in-person versus telehealth format of PE, leading to the conclusion that the two modalities are likely equivalent [30,33,34]. Our findings show that although these modalities might be equivalent on average, they likely are not equivalent at the individual level. A set of individual characteristics related to mental health disparities and differential access to care—including age, race, disability status, disadvantaged socioeconomic background, and rural status [46,47,48,50,51,53,54,55]—seem to also contribute to differential response to PE modalities. Optimizing treatment assignment based on these characteristics may contribute to the advancement of mental health equity and thus maximize mental health outcomes.

It is worth noting that the estimated optimal individualized treatment rule was significantly superior to universal assignment to telehealth but not to in-person PE. There are two possible explanations for this finding. First, the lack of significance might be due to low statistical power, which did not allow us to identify an existing difference. Second, it is likely that assigning everyone to in-person PE will lead to a reduction in PTSD symptoms similar to individualizing assignment to a PE modality based on their characteristics. In other words, although telehealth may not be universally beneficial to everyone, in-person PE may be. If this is further corroborated, one might wonder: if in-person PE is either equally or more effective than telehealth for individuals, why use the individualized treatment rule and not just universally assign everyone to in-person PE? In-person therapy involves barriers for certain clients, such as time constraints, the need for transportation, increased cost, and possibly perceived stigma [78,79,80]. It also requires greater availability of office space and other resources for clinics/hospitals, and thus higher health service costs [81]. Therefore, there are clear benefits of utilizing the telehealth modality when equally or more effective for an individual.

An important strength of the approach used in this study is the rigorous data-driven estimation of an easily implemented in practice decision rule that can aid clinical decision-making regarding the assignment to the two PE modalities. Such decisions are typically based entirely on clinical judgment and/or patient preferences [45]. This has important disadvantages associated with human error and biases, leading to suboptimal decisions [82,83,84]. Combining multiple baseline characteristics simultaneously using a flexible and rigorous machine-learning-based approach can overcome the downsides of human decision-making [45]. Previous research has provided evidence for the superiority of individualized treatment rules over clinical judgment [82,83,84].

Upon further rigorous validation of our estimated rule in larger samples, more diverse veteran populations, and using external validation procedures, such a rule can be easily adopted in clinical settings treating women veterans with MST-related PTSD. This can be done by using the baseline characteristics used to construct the rule—i.e., age, race, disability status, socioeconomic status, rural vs. urban status, and baseline PTSD level—as input to a computer program (such as Microsoft Excel v. 16.89.1) implementing the decision rule equation provided in the results section using the estimated coefficients [45]. In other words, inserting a new client’s characteristics into the function would yield a recommended assignment to either in-person or telehealth PE. This way, clinicians can make more informed decisions about which modality (in-person or telehealth) is best suited for each patient based on their specific characteristics, leading to more personalized care. In addition, understanding which patients benefit most from each modality allows healthcare systems to allocate resources more efficiently [85]. This can lead to better scheduling, reduced wait times, and maximized clinician productivity. More importantly, by directing patients to the most appropriate mode of therapy, this approach can improve treatment outcomes [45].

Although this rule cannot be directly applied to other populations as is, this approach has far-reaching implications not only for women veterans treated for MST-related PTSD but also for other populations facing barriers to care. Telehealth is non-inferior to in-person psychotherapy on average for a variety of populations and mental health concerns [86], but response to each might differ at the individual level. The individual characteristics we considered are relevant to most treatment-seeking populations, and thus, individualized treatment rules with the same prescriptive factors can be examined to optimize assignment to telehealth and in-person modalities for any set of psychotherapies and clinical populations. Results from our study might generalize to women veterans with other mental concerns who face similar systemic barriers due to the male-dominated culture in VA settings [16]. This is an important question to consider in future research. Comparing a rule with the addition of gender as a prescriptive factor to the rule that we estimated will allow us to assess whether this characteristic further differentiates benefits between the two treatment modalities. Regarding PTSD more generally, initiation of PTSD treatment, especially as exposure-heavy as PE is, might be particularly challenging even outside of the VA setting [87]. Therefore, applying a rule to optimize allocation between the two PE modalities that takes into account individual characteristics may facilitate initial engagement and continuation of treatment.

Several limitations of the present study need to be noted. First, the sample size of the current study was small, which has two implications: (1) It was not possible to split the sample into a training and testing sample to estimate the out-of-sample performance of the individualized treatment rule, which could increase the possibility of overfitting. However, to address the problem of overfitting, we used the leave-one-out cross-validation method, which is appropriate for smaller sample sizes [76]. (2) The small sample size could have led to increased Type II error, which might explain the lack of significant difference between the individualized rule and the one-size-fits-all rule of universal assignment to in-person PE. Second, most participants identified as Black or White, with low representation of other races. Therefore, this rule may not capture well the needs of treatment-seeking individuals with other racial backgrounds. Research is needed to validly examine this rule in more racially diverse populations. Third, our study considered only a portion of individual characteristics that might relate to barriers to care access. For example, LGBTQIA+ community members who experience high rates of trauma, discrimination, and minority stress face critical barriers to accessing health and mental health services [88]. Therefore, this is an important characteristic to consider in future research. Fourth, although the focus of our study was women veterans, further research is needed with male veterans with MST-related PTSD. Men with MST may face different barriers compared to women, including subjective experiences of shame resulting from stigma related to masculine stereotypes and gendered beliefs about rape, which may result in MST underreporting and different patterns of treatment access and response [89,90]. Fifth, one important variable that might impact response to PE modality that was not considered in the present study is patient preferences. Patient preference towards one modality versus the other may play a role in how one responds to treatment [16]. For example, someone who was forced to complete treatment via telehealth but had a strong preference for in-person therapy might put in less effort, be less compliant in homework completion, or discontinue treatment altogether. Thus, this may be an important variable to take into account for clinical decision-making. This is particularly true in the context of MST, which is often closely linked to feelings of powerlessness and lack of control, because of stripping the survivor’s autonomy and agency during the assault, as well as the fear of retaliation [91]. Taking patient preference into account and encouraging personal choice can increase empowerment of survivors and improve outcomes overall. Future research on individualized assignment between the two PE modalities should incorporate patient preferences in the set of prescriptive factors. Sixth, although the current clinical trial had relatively lenient exclusion criteria and included diverse profiles of client presentations, increasing the representativeness of the sample, individuals with high suicide risk were excluded. This might induce selection bias, leading to the estimated rule not generalizing to a population with more severe symptomatology. Additional research is needed in this population, which could be facilitated by studies using real-world data that have higher external validity and can address some of the limitations of clinical trials.

## 5. Conclusions

To our knowledge, this study was the first to implement a machine-learning precision medicine approach to estimate an individualized treatment rule to optimize assignment between in-person and telehealth PE for women veterans with MST-related PTSD. This rule leveraged easily accessible individual characteristics associated with systemic barriers to accessing care to maximize benefit from treatment. Findings highlight that although the two treatment modalities are equivalent on average, treatment response may differ at the individual level. The estimated individualized treatment rule performed significantly better than universally assigning everyone to telehealth. The estimated rule did not perform significantly better than universally assigning everyone to in-person. Upon further validation in larger and more diverse samples, such a rule can be applied in practice to aid clinical decision-making and personalization of treatment assignment to individual needs.

## Figures and Tables

**Table 1:** Study characteristics.

	Overall		Telehealth PE ^1^		In-Person PE ^1^			% Missing
	Mean	SD	Mean	SD	Mean	SD	*p*	
**Baseline measures**								
Age	42.80	11.61	40.65	11.99	45.02	10.86	0.037	0.00
PCL-5	52.74	12.81	53.16	13.17	52.30	12.52	0.712	0.82
**Outcome**								
6-month PCL-5	31.63	21.84	33.29	21.02	30.10	22.72	0.519	35.25
	**N**	**%**	**N**	**%**	**N**	**%**		
Race							0.929	0.00
White	34	27.87	18	29.03	16	26.67		
Black	79	64.75	39	62.90	40	66.67		
Other race	9	7.38	5	8.06	4	6.67		
Disability status							0.755	2.46
Yes	76	63.87	37	61.67	39	66.10		
No	43	36.13	23	38.33	20	33.90		
Rural status							1.000	0.00
Yes	42	34.43	21	33.87	21	35.00		
No	80	65.57	41	66.13	39	65.00		
Annual income								0.82
USD 0–<USD 10,000	10	8.33	5	8.20	5	8.47	0.142	
USD 10,000–< USD 15,000	5	4.17	5	8.20	0	0.00		
USD 15,000–< USD 20,000	9	7.50	5	8.20	4	6.78		
USD 20,000–< USD 25,000	12	10.00	6	9.84	6	10.17		
USD 25,000–< USD 35,000	23	19.17	10	16.39	13	22.03		
USD 35,000–< USD 50,000	36	30.00	14	22.95	22	37.29		
USD 50,000–< USD 75,000	10	8.33	8	13.11	2	3.39		
USD 75,000 or more	15	12.50	8	13.11	7	11.86		

^1^ Prolonged exposure.

**Table 2:** Performance of the optimal individualized treatment rule: Estimated PCL-5 level under the estimated rule and comparison with fixed, one-size-fits-all rules.

Parameter	Estimate (95% CI)
*V*d_opt_ ^1^	17.83 (3.90, 31.76)
*V*d_opt_ − *V*_Telehealth_ ^2^	−14.55 (−27.24, −1.86)
*V*d_opt_ − *V*_In-person_ ^3^	−11.86 (−25.83, 2.12)

^1^ Vd_opt_ = Mean PCL-5 score under the estimated optimal rule. ^2^ Vd_opt_ − V_Telehealth_ = Difference in mean PCL-5 score between the estimated optimal rule and the one-size-fits-all rule of universal assignment to telehealth PE. ^3^ Vd_opt_ − V_In-person_ = Difference in mean PCL-5 score between the estimated optimal rule and the one-size-fits-all rule of universal assignment to in-person PE.

## Data Availability

Data are available upon request from the principal investigator.
